# Comprehensive Expression of Wnt Signaling Pathway Genes during Development and Maturation of the Mouse Cochlea

**DOI:** 10.1371/journal.pone.0148339

**Published:** 2016-02-09

**Authors:** Ruishuang Geng, Teppei Noda, Joanna F. Mulvaney, Vincent Y. W. Lin, Albert S. B. Edge, Alain Dabdoub

**Affiliations:** 1 Biological Sciences, Sunnybrook Research Institute, Toronto, Ontario, Canada; 2 Department of Otolaryngology—Head and Neck Surgery, University of Toronto, Toronto, Ontario, Canada; 3 Department of Otology and Laryngology, Harvard Medical School, Boston, Massachusetts, United States of America; 4 Department of Laboratory Medicine and Pathobiology, University of Toronto, Toronto, Ontario, Canada; Texas A&M University, UNITED STATES

## Abstract

**Background:**

In the inner ear Wnt signaling is necessary for proliferation, cell fate determination, growth of the cochlear duct, polarized orientation of stereociliary bundles, differentiation of the periotic mesenchyme, and homeostasis of the stria vascularis. In neonatal tissue Wnt signaling can drive proliferation of cells in the sensory region, suggesting that Wnt signaling could be used to regenerate the sensory epithelium in the damaged adult inner ear. Manipulation of Wnt signaling for regeneration will require an understanding of the dynamics of Wnt pathway gene expression in the ear. We present a comprehensive screen for 84 Wnt signaling related genes across four developmental and postnatal time points.

**Results:**

We identified 72 Wnt related genes expressed in the inner ear on embryonic day (E) 12.5, postnatal day (P) 0, P6 and P30. These genes included secreted Wnts, Wnt antagonists, intracellular components of canonical signaling and components of non-canonical signaling/planar cell polarity.

**Conclusion:**

A large number of Wnt signaling molecules were dynamically expressed during cochlear development and in the early postnatal period, suggesting complex regulation of Wnt transduction. The data revealed several potential key regulators for further study.

## Introduction

Wnt signaling is an essential regulator of embryonic development and homeostasis [1]. Given that Wnt signaling has a role in organogenesis and in stem cell renewal, it is an excellent candidate for inducing regeneration following damage to sensory organs [2–4]. Activation of canonical Wnt signaling in the inner ear during development and at neonatal time points results in proliferation of prosensory cells and supporting cells, underlining its potential as a route to hearing restoration [2]; however, this capacity for β-catenin mediated proliferation does not continue past neonatal stages. Identification of Wnt signaling components in the inner ear across developmental time points, is essential for both understanding its varied roles in development, and exploring its regenerative potential.

The Wnt signaling network has three primary pathways: canonical β-catenin mediated Wnt signaling, non-canonical planar cell polarity (PCP) Wnt signaling and Wnt/calcium signaling. The canonical pathway is transduced by binding of Wnt ligands to Frizzled and Lrp receptors to sequester the protein kinase GSK3β, preventing it from targeting β-catenin for destruction [1]. The non-canonical Wnt PCP pathway acts to provide directionality to individual cells and groups of cells, by generating polarized distribution of intracellular and extracellular components on individual cells. Secreted Wnt molecules that bind to Frizzled and Ryk receptors provide directional cues [1]. The Wnt/calcium pathway is activated by binding of Wnt ligands to Frizzled receptors, which leads to activation of intracellular signaling molecules diacylglycerol (DAG), inositol trisphosphate (IP3) and release of calcium ions to activate calcium signaling effectors such as protein kinase C (PKC) and calcium/calmodulin kinase II (CaMKII) [5]. Given that extracellular context and the composition of intracellular components will influence which path Wnt signaling will take [1], characterization of the specific Wnt signaling components expressed in any given tissue is required to allow manipulation of this complex network.

Both canonical and PCP signaling are involved in formation of the mammalian inner ear. Canonical Wnt signaling is active in early stages of mammalian otic development [6], where it specifies the size of the placode [7] and functions to compartmentalize otic precursors in the otocyst between dorsal fate (vestibular system) and ventral fate (cochlea) [8] [9]. Later, from E12.5 and onwards, when the cochlear duct has emerged from the otocyst, canonical Wnt signaling regulates cell fate decisions in the sensory epithelium [10, 11]. The sensory epithelium is a highly ordered, stratified structure consisting of one row of inner hair cells, and three rows of outer hair cells. Inner hair cells are segregated from outer hair cells by two intervening rows of pillar cell supporting cells, and each row of outer hair cells alternates with a row of Deiters’ cell supporting cells. The precise arrangement and number of hair cells and supporting cells is essential for optimal hearing. Inhibition of Wnt signaling through use of pharmacological agents or loss of β-catenin results in a failure of hair cells to differentiate [10, 11]. Subsequently, once hair cells have differentiated, Wnt PCP signaling orients the stereociliary bundles in a uniform direction [12] [13] and mediates elongation of the cochlear duct [13].

Previously, comprehensive screens for Wnt related genes in the developing chicken inner ear [14], and single cell analysis of the neonatal sensory epithelium [15], have provided valuable insight. A screen focusing on Wnt components expressed throughout the mammalian cochlea across several developmental time points, would complement these studies and identify previously uncharacterized components of Wnt signaling in the ear that can be targeted for further analysis.

Here we present gene expression profiling of 72 Wnt signaling related genes expressed in the cochlea at embryonic, perinatal, juvenile and adult stages of development, and spatiotemporal localization of three secreted Wnt antagonists.

## Materials and Methods

### Cochlear dissection and tissue preparation

Care and euthanasia of CD-1 mice (Charles River laboratory) used in this study was approved, and conformed to IACUC regulations, by Sunnybrook Research Institute Animal Care Committee. Mice were maintained in habitat enriched (Bioserv mouse Igloo) isolation cages with automated watering under 12 hour light/12 hour dark cycles at 21°C. Fewer than five adult animals were housed in each cage; cages were changed weekly. Water and food were available *ad libitum*. Mice were fed the standard irradiated diet, supplied by Harlan. Adult mice were euthanized by CO2, and neonatal mice by decapitation. Cochlear sensory epithelium with stria vascularis and Reissner’s membrane were dissected free from nerve tissue on E12.5, P0, P6 and P30. Tissue from 10 to 12 cochleae were pooled at each stage and stored in RNA*later* (Qiagen). RNA was extracted within 24 hours.

### RNA extraction

Total RNA was extracted using RNeasy mini kit (Qiagen) with on column DNase treatment according to the manufacturer’s instructions. RNA concentration was analyzed using Qubit fluorometer, samples containing more than 3% DNA were discarded.

### cDNA library preparation

cDNA was prepared from 500 ng of RNA using the RT first strand kit (Qiagen, Cat # 330401), during this process residual genomic DNA was eliminated. Quality of the reverse transcription reactions was assessed using a genomic DNA control plate (Qiagen, PAMM-999ZC-1). No amplification of genomic DNA was detected.

### RT^2^-profiler PCR array

In order to perform PCR, for each time point: each reverse transcription reaction product was mixed with RT SYBR Green OX qPCR master mix (Qiagen, 330522) and aliquoted into the PCR arrays. Each 96-well plate contained 84 Wnt signaling related genes, one genomic DNA quality control, three reverse transcription controls, three PCR controls and five housekeeping genes including *Actb*, *B2m*, *Gapdh*, *Gusb*, and *Hsp90ab1* (Qiagen, PAMM043ZC-12). A complete list of assays contained in the plate is available from the manufacturer (Qiagen, PAMM043ZC-12). Thermal cycling was performed using StepOnePlus instrument (Applied Biosystems) under the following conditions: 10 minutes at 95°C and 40 cycles of 15 seconds at 95°C and one minute at 60°C. Two biological repeats were performed for each time point.

Quality control measures for all plates:

The genomic DNA control well exhibited no detectable amplification.

Positive PCR control wells attained a Ct value of 20±2, demonstrating that no PCR inhibitory contamination was present.

Reverse transcription control wells achieved a Ct value within five cycles of the positive PCR control assays, demonstrating efficiency of transcription.

Four housekeeping gene assays reached a Ct value of around 20. These Ct values were consistent across all the time points and across biological replicates.

Analysis:

Melting curves of all wells were examined and only data from wells with a single peak after melting were included in analyses. Ct values of the internal control genes were averaged (Ct = 20.1) and used for normalization in order to calculate fold changes in gene expression.

### Real-Time Quantitative PCR

RT qPCR was performed using *Taqman* probes and reagents following manufacturer’s instructions, *Wif1* (Mm00442355_m1); *Wisp1* (Mm01200484_m1); *Dkk3* (Mm00443800); *Kremen1* (Mm00459616_m1); *Sfrp1* (Mm00489161_m1); *Wnt11* (Mm00437328_m1); *Atoh1* (Mm00476035_s) and *Sox2* (Mm00488369_s1) Each assay was analyzed in triplicate.

### Preparation of riboprobes

Probe templates for *Dkk3* and *Sfrp3* were generated as previously described [16] [17]. *Sfrp1* was cloned into pBluescript II SK (+) from cDNA obtained from embryonic mouse cochleae. Sfrp1 primers: Forward GAAATGCAAAGTCTCCTGAGTG, Reverse AGGCTTAGGCTATAAAGTGGAG. DIG labeled riboprobes were prepared as previously described [9], *Sfrp1* template plasmid was digested with SpeI and transcribed with T7 RNA polymerase. Riboprobes were purified on G-50 micro columns (GE Healthcare).

### RNA *In situ* hybridization

Embryonic or postnatal cochleae were dissected and fixed in 4% paraformaldehyde. P6 and P30 cochleae were decalcified by pH 5.2 10% EDTA in RNA*later* [18] for 24 hours and 48 hours, respectively. After cochleae were cryosectioned and mounted on positively charged glass slides (Superfrost Plus Microscope Slides, Fisher Scientific), RNA *in situ* hybridization was performed as previously described [9]. Briefly, the specimens were permeabilized with 2μg/ml proteinase K, re-fixed in 4% paraformaldehyde and acetylated. Hybridization was performed at 55 to 70°C. Imaging was performed using an Axio Scope A1 microscope fitted with an Axiocam (Carl Zeiss).

### Immunohistochemistry

Immunohistochemistry was performed subsequent to *in situ* hybridization as previously described [19]. Primary antibody: Rabbit Myosin-7a (Myo7a) (1:1000; Proteus Biosciences) and Goat Prox1 (1:500; R&D Systems). Imaging was performed using an Axio Scope A1 microscope fitted with an Axiocam (Carl Zeiss).

## Results and Discussion

### Multiple Wnt signaling related genes are expressed in the developing and postnatal cochlea

#### Identification of genes expressed at high and low levels in the cochlear duct

To obtain a comprehensive profile of Wnt signaling related gene expression in the cochlea, we used a commercially available RT^2^ Profiler™ PCR Array (Qiagen), pre-designed to detect 84 Wnt signaling related genes. We performed analysis at four time points, embryonic day (E) E12.5, postnatal day (P) 0, P6 and P30. These stages were selected to reflect the differing levels of Wnt related activity as development progresses. On E12.5, Wnt signaling is highly active in the prosensory epithelium as shown by Wnt reporter activity [10], and cells respond to activation of β-catenin by proliferating [10] [11]. As development progresses, endogenous levels of Wnt reporter activity in the cochlear epithelium are reduced, such that low levels are reported in the base of the cochlear duct on E14.5 [10], and by E17.5, activity is restricted to pillar cells, inner phalangeal cells and lateral supporting cells at low levels [10]. Pharmacological disruption of Wnt signaling on E16.5 [10], or β-catenin ablation in Gfi1 expressing hair cells resulted in no discernable phenotype [11], indicating that endogenous levels of Wnt signaling are down regulated as development proceeds. On P0, the cochlear developmental program is advanced, and stimulation of the canonical Wnt pathway resulted in a more modest mitogenic response [2]. By P6 endogenous canonical Wnt signaling is no longer active and the ability of cells in the sensory region to respond to ectopic Wnt activity in the sensory region of the cochlea is attenuated [2]; however, Wnt signaling involved in homeostasis of the stria vascularis remains active [20]. P30 was chosen as a time point where developmental genes are likely to be down regulated.

Expression profiling was performed on the entire cochlear duct. This was done to capture expression of genes involved in development and maintenance of the stria vascularis and cochlear morphology, in addition to those involved in sensory development. Furthermore, Wnt signaling relies on secreted Wnts, secreted Wnt potentiators and secreted antagonists; all of these molecules can be expressed at a distance from cells expressing Wnt receptors. While studies focusing exclusively on determining gene expression in cells of the sensory region are important and informative, an advantage of using the whole organ is that it allows detection of genes encoding secreted molecules that diffuse from the nonsensory regions of the cochlea; this is a pertinent consideration when expression profiling is targeted for paracrine cell signaling.

RT^2^ Profiler™ PCR Array assays were performed using RNA extracted from 10–12 cochleae harvested on E12.5, P0, P6 and P30. Relative levels of mRNA were calculated based on cycle threshold number (Ct) and fold change calculated based on ΔCt. Initial analysis was to determine presence or absence of each gene at each developmental stage. Genes were grouped based on their Ct values (Table 1). Genes with a Ct value of less than 25 were classed as abundantly expressed, while genes with a Ct value of between 25 and 30 inclusive, were assessed as having low levels of expression. We discounted genes with a Ct value over 30 as these expression levels are too low to be reliably indicative of biologically active levels of expression.

**Table 1:** Wnt signaling related genes expressed in developing and mature mouse cochlea detected by RT^2^ Profiler PCR Array.

E12.5		P0		P6		P30	
Ct<25	Ct = 25–30	Ct<25	Ct = 25–30	Ct<25	Ct = 25–30	Ct<25	Ct = 25–30
*Aes*	*Daam1*	*Aes*	*Bcl9*	*Aes*	*Bcl9*	*Aes*	*Bcl9*
*Apc*	*Dkk3*	*Apc*	*Daam1*	*Apc*	*Daam1*	*Apc*	*Daam1*
*Axin1*	*Dvl1*	*Axin1*	*Dkk3*	*Axin1*	*Dvl1*	*Axin1*	*Dvl2*
*Axin2*	*Fbxw4*	*Axin2*	*Dvl1*	*Axin2*	*Dvl2*	*Axin2*	*Fbxw4*
*Bcl9*	*Frat1*	*β-Trc*	*Dvl2*	*β-Trc*	*Fbxw4*	*β-Trc*	*Frat1*
*β-Trc*	*Fzd5*	*Ccnd1*	*Ep300*	*Ccnd1*	*Frat1*	*Ccnd1*	*Fzd3*
*Ccnd1*	*Fzd8*	*Ccnd2*	*Fbxw4*	*Ccnd2*	*Fzd5*	*Ccnd2*	*Fzd5*
*Ccnd2*	*Fzd9*	*Csnk1a1*	*Frat1*	*Csnk1a1*	*Fzd8*	*Csnk1a1*	*Fzd8*
*Csnk1a1*	*Myc*	*Csnk2a1*	*Fzd5*	*Csnk2a1*	*Myc*	*Csnk2a1*	*Lef1*
*Csnk2a1*	*Nfatc1*	*Ctbp1*	*Fzd8*	*Ctbp1*	*Nfatc1*	*Ctbp1*	*Myc*
*Ctbp1*	*Porcn*	*β-Catenin*	*Jun*	*β-Catenin*	*Porcn*	*β-Catenin*	*Nfatc1*
*β-Catenin*	*Ppard*	*Ctnnbip1*	*Myc*	*Ctnnbip1*	*Prickle1*	*Ctnnbip1*	*Porcn*
*Ctnnbip1*	*Prickle1*	*Dab2*	*Nfatc1*	*Dab2*	*Pygo1*	*Dab2*	*Ppard*
*Dab2*	*Pygo1*	*Dixdc1*	*Nlk*	*Dixdc1*	*Sox17*	*Dixdc1*	*Prickle1*
*Dixdc1*	*Sfrp3*	*Fbxw11*	*Porcn*	*Dkk3*	*Tcf7l1*	*Dkk3*	*Pygo1*
*Dvl2*	*Sox17*	*Fzd1*	*Ppard*	*Ep300*	*Tle1*	*Dvl1*	*Sfrp2*
*Ep300*	*Tcf7*	*Fzd2*	*Prickle1*	*Fbxw11*	*Wnt11*	*Ep300*	*Sfrp4*
*Fbxw11*	*Tcf7l1*	*Fzd3*	*Pygo1*	*Fzd1*	*Wnt16*	*Fbxw11*	*Sox17*
*Fzd1*	*Wif1*	*Fzd4*	*Sox17*	*Fzd2*	*Wnt2*	*Fzd1*	*Tcf7*
*Fzd2*	*Wisp1*	*Fzd6*	*Tcf7*	*Fzd3*	*Wnt2b*	*Fzd2*	*Tcf7l1*
*Fzd3*	*Wnt11*	*Fzd7*	*Tle1*	*Fzd4*	*Wnt4*	*Fzd4*	*Tle1*
*Fzd4*	*Wnt2b*	*Fzd9*	*Wif1*	*Fzd6*	*Wnt5b*	*Fzd6*	*Vangl2*
*Fzd6*	*Wnt4*	*Gsk3β*	*Wisp1*	*Fzd7*	*Wnt6*	*Fzd7*	*Wif1*
*Fzd7*	*Wnt5b*	*Kremen1*	*Wnt11*	*Fzd9*	*Wnt7a*	*Fzd9*	*Wisp1*
*Gsk3β*	* Wnt7a*	*Lef1*	*Wnt2b*	*Gsk3β*	*Wnt7b*	*Gsk3β*	*Wnt11*
*Jun*	* Wnt7b*	*Lrp5*	*Wnt4*	*Jun*	*Wnt9a*	*Jun*	*Wnt16*
*Kremen1*	* Wnt9a*	*Lrp6*	*Wnt5b*	*Kremen1*		*Kremen1*	*Wnt4*
*Lef1*		*Mapk8*	*Wnt6*	*Lef1*		*Lrp5*	*Wnt5b*
*Lrp5*		*Nkd1*	*Wnt7a*	*Lrp5*		*Lrp6*	*Wnt6*
*Lrp6*		*RhoA*	*Wnt7b*	*Lrp6*		*Mapk8*	*Wnt7a*
*Mapk8*		*RhoU*	*Wnt9a*	*Mapk8*		*Nkd1*	*Wnt7b*
*Nkd1*		*Ruvbl1*		*Nkd1*		*Nlk*	*Wnt9a*
*Nlk*		*Sfrp1*		*Nlk*		*RhoA*	
*RhoA*		*Sfrp2*		*Ppard*		*RhoU*	
*RhoU*		*Sfrp3*		*RhoA*		*Ruvbl1*	
*Ruvbl1*		*Tcf7l1*		*RhoU*		*Sfrp1*	
*Sfrp1*		*Vangl2*		*Ruvbl1*		*Sfrp3*	
*Sfrp2*		*Wnt5a*		*Sfrp1*		*Wnt2b*	
*Tle1*				*Sfrp2*		*Wnt5a*	
*Vangl2*				*Sfrp3*			
*Wnt5a*				*Sfrp4*			
				*Tcf7*			
				*Vangl2*			
				*Wif1*			
				*Wisp1*			
				*Wnt5a*			

**Fig 1:**
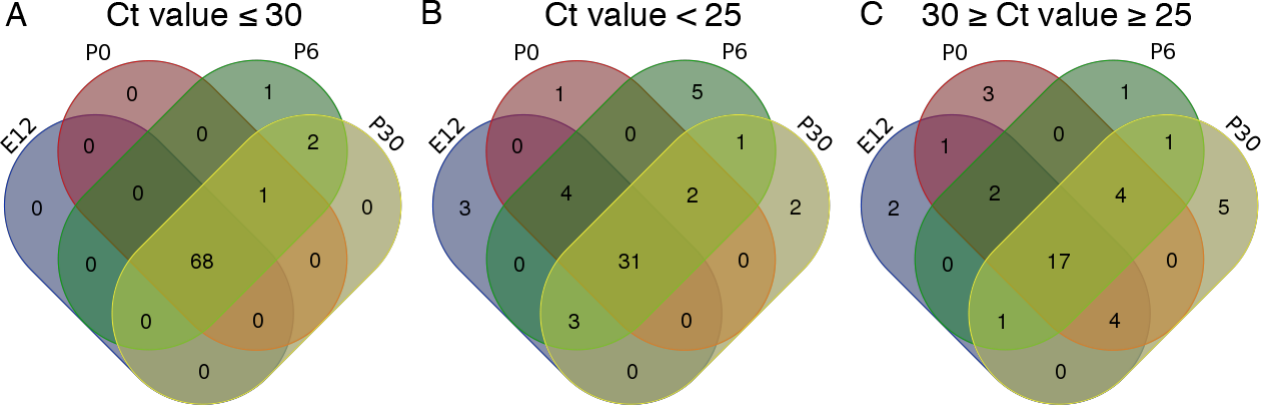
Expression of 72 Wnt signaling related genes was detected in the cochlea. (A) A Venn diagram illustrating the temporal distribution of screened genes that reached cycle threshold in 30 or fewer cycles. (B) A Venn diagram, illustrating the temporal distribution of genes that reached cycle threshold in fewer than 25 cycles. (C) A Venn diagram, illustrating the temporal distribution of genes that reached cycle threshold in between 25 and 30 (inclusive) cycles.

In summary, of the 84 Wnt signaling related genes tested, 72 were detected (Table 1); 68 had a Ct value of 30 or below in all samples (Table 1, Fig 1A); one gene (*Wnt6*) was detected on P0, P6 and P30 but not E12.5; two genes (*Sfrp4* and *Wnt16*) were expressed on P6 and P30 only (Table 1, Fig 1A, 1B and 1C); and one gene (*Wnt2*) was expressed only on P6 (Table 1, Fig 1A, 1B and 1C). 53 genes were found with a Ct value of less than 25 at least at one time point (Table 1, Fig 1B). Of these genes, 31 were found at all stages (Table 1 Fig 1B); four (*Lef1*, *Frizzled 3*, *Vangl2*, *Sfrp2*) were detected in E12.5, P0 and P6 (Table 1, Fig 1B); three (*Ep300*, *Jun*, *Nlk*) were expressed at relatively high levels on E12.5, P6 and P30 but low levels on P0 (Table 1 Fig 1B); two (*Sfrp3*, *Frizzled 9*) were highly expressed on P0, P6 and P30 but low levels on E12.5 (Table 1 Fig 1B); one gene (*Dkk3*) was highly expressed on P6 and P30 and expressed at low levels on E12.5 and P0; three genes (*Bcl9*, *Tle1*, *Dvl2*) were expressed at high levels on E12.5 and low levels at all other stages; one gene (*Tcf7l1*) was highly expressed on P0; five genes (*Sfrp4*, *Wisp1*, *Wif1*, *Tcf7*, *Ppard*) were expressed at high levels on P6; two genes (*Dvl1* and *Wnt2b*) were expressed at higher levels on P30. Genes that were amplified with a Ct value of greater than 30, or exhibited a sub-optimal melt curve across all samples, were discounted (*Wnt1*, *Wnt3a*, *Wnt3*, *Wnt8a*, *Wnt8*, *Wnt10a*, *Fgf4*, *Mmp7*, *Dkk1*, *Pitx2*, *FoxN1 and Fosl1)*.

Having identified that 72 Wnt signaling related genes were expressed with a Ct value of 30 or below on E12.5, P0, P6 or P30, we examined the distribution of sub-classes of Wnt signaling related genes at each stage in more detail.

#### Expression of extracellular Wnt components

Of the 19 mammalian Wnt genes, only *Wnt5a* was found to have a Ct value of less than 25 across all time points, and *Wnt2b* at Ct less than 25 on P30. *Wnt11*, *Wnt7b*, *Wnt5b*, *Wnt4*, *Wnt7a*, and *Wnt9a* were found at lower levels (Ct value of between 25 and 30) at all time points, *Wnt6* was not detected on E12.5 but was detected at low levels on P0, P6 and P30, and *Wnt16* only on P6 and P30 (Table 1, Table 2). This is consistent with previous reports that place Wnt5a and Wnt7a in the PCP pathway during development [12, 13]. As yet, Wnt molecules responsible for activating canonical Wnt signaling in the cochlea have not been reported; we show that the expression of *Wnt4*, *Wnt5b*, *Wnt7b* and *Wnt11* is coincident with sensory development providing candidate Wnt molecules for further investigation. Given their late onset of expression, *Wnt2b* and *Wnt6* are likely not involved in developmental processes.

**Table 2:** Changes in Wnt signaling related gene expression levels during cochlear development and maturation analyzed by RT^2^ Profiler PCR Array.

P0 vs E12.5				P6 vs P0				P30 vs P6			
Up	Fold change	Down	Fold change	Up	Fold change	Down	Fold change	Up	Fold change	Down	Fold Change
*Wnt6*	+10	*Ccnd1*	-3.6	*Wisp1*	+8.0	*Wnt11*	- 2.5	*Wnt2b*	+3.4	*Sfrp2*	-28.6
*Dkk3*	+6.2	*Jun*	-3.5	*Sfrp3*	+4.1			*Dkk3*	+2.8	*Wif1*	-4.4
*Fzd9*	+5.3	*Dvl1*	-2.7	*Wif1*	+3.2			*Wnt6*	+2.8	*Myc*	-4.2
*Wif1*	+4.8	*Wnt4*	-2.4	*Wnt4*	+3.2			*Dvl1*	+2.3	*Lef1*	-3.2
*Wnt5a*	+4.1	*Fzd7*	-2.2	*Fzd5*	+3.1					*Sfrp1*	-3.2
*Wisp1*	+4.0	*Ctbp1*	-2.0	*Kremen1*	+3.0					*Ccnd1*	-3.1
*Tcf7*	+2.8	*Lef1*	-2.0	*Sox17*	+2.6					*Vangl2*	-3.1
*RhoU*	+2.6	*Mapk8*	-2.0	*Dvl1*	+2.5					*Fzd1*	-2.8
*Wnt9a*	+2.1			*Fzd9*	+2.3					*Ccnd2*	-2.6
*Sfrp3*	+2.0			*Wnt5a*	+2.3					*Wnt4*	-2.6
				*Fzd6*	+2.2					*Nkd1*	-2.4
				*Nlk*	+2.1					*Tcf7*	-2.3
				*Porcn*	+2.1					*Wnt5b*	-2.2
										*Dab2*	-2.1
										*Prickle1*	-2.1
										*Tle1*	-2.0

*Frizzleds 1*, *2*, *3*, *4*, *6*, and *7* were detected with a Ct value of less than 25 at all time points. *Frizzled 9* was expressed at low levels on E12.5 and most highly expressed on P0, P6 and P30. *Frizzleds 5* and *8* were expressed at low levels at all time points. This is consistent with previous reports that demonstrate Frizzled 4 in the stria vascularis [20], and Frizzled 1, 2, 3, 4 and 6 in the sensory epithelium [2, 20, 21].

Canonical Wnt receptors *Lrp5* and *6* were found to have a Ct value of less than 25 at all time points. This is expected since canonical Wnt signaling requires Lrp5/6 [1]. Wnt PCP transmembrane molecule *Vangl2* was expressed at Ct lower than 25 on E12.5, P0 and P6, but was undetected on P30 and *Prickle1* at low levels at all time points. Vangl2 has been shown to function in stereociliary bundle orientation during development [22], while Prickle is a well characterized transmembrane PCP component [1].

Seven genes encoding Wnt antagonists had a Ct value of less than 25. *Kremen1* was detected with a Ct of less than 25 at all time points, whereas of the two Dkk molecules tested, *Dkk3* was detected with a Ct value of between 25 and 30 on E12.5 and P0, with expression increasing such that on P6 and P30, it appeared with a Ct value of less than 25; *Dkk1* was undetected at all stages. *Sfrp1* was found to be highly expressed at all time points. *Sfrp2* was detected on E12.5, P0 and P6 at fewer than 25 cycles. *Sfrp3* was detected at fewer than 25 cycles at P0, P6 and P30. *Sfrp4* was detected at P6 and P30 only. Finally *Wif1* was found with a Ct value of less than 25 only at P6 and P30, on E12.5 and P6, expression was detected at lower levels.

#### Expression of intracellular components of the Wnt signaling network

Having examined extracellular members of the Wnt signaling network, we inspected expression of genes encoding intracellular components of Wnt signaling. *Dishevelled 1* was detected on E12.5, P0 and P6 with a Ct value of 30 or less, and was highly expressed on P30. *Dvl2* was detected with a Ct less than 25 on E12.5, and expressed at low levels on P0, P6 and P30. Disheveled adaptor protein *Dixdc1* was found to have a Ct value of less than 25 at all time points. Both *Dishevelled* homologues have been implicated in PCP in the cochlea [2]. Components of Wnt PCP found at high levels included *RhoA* and *RhoU* at all time points; *Daam1* was expressed at low levels at all time points.

As expected, intracellular components of canonical Wnt signaling involved in destruction of β-catenin, and *β-catenin* itself were maintained at high levels at all stages of development (*Gsk3β*, *Axin1*, *Axin2*, *Apc*, *Ck1α*, *Ck2*, *Nkd1*). Ubiquitin ligases *β-trcp1* and *2*; *Ctnnbip1* (ICAT) and *Aes—*negative regulators of β-catenin/TCF4 interaction; co-repressor *CTBP1*, and *Dab2* an LRP inhibitor, were all found with a Ct value lower than 25 at all time points. *Lef1* a component of the β-catenin activated transcriptional complex was present at high levels on E12.5, P0 and P6 and at low levels on P30. *Tle1 and Bcl9*, nuclear β-catenin co-activators, were only highly expressed on E12.5. The presence of intracellular components of canonical Wnt signaling is consistent with their known biological roles. The down regulation of *Tle1 and Bcl9* after E12.5 is noteworthy, since it coincides with the decline in the response of prosensory or supporting cells to stabilization of β-catenin [2].

In summary, we identified temporal expression of 72 Wnt signaling related genes in the cochlea at stages E12.5, P0, P6 and P30. The results of this screen underline the importance of Wnt signaling activity during development of the inner ear, providing novel information about the dynamics of Wnt related gene expression as the cochlea matures, and identifying components of the Wnt network that should be investigated in future work.

### Wnt signaling related genes are dynamically expressed as the cochlea matures

#### Expression of several Wnt signaling related genes was dependent on developmental time point

The Ct values of the data set clearly indicated that the expression levels of individual genes varied from one stage to another. Fold change in gene expression was calculated between different time points using ΔCt method (Table 2). We identified several genes that showed a greater than two fold change between time points (Table 2). In the time period between E12.5 and P0 Wnt signaling is at its most active, stimulating cells to proliferate, differentiate and undergo morphogenetic movement as the prosensory region is organized. On P0 canonical Wnt signaling is less active, but Wnt PCP continues to be required for orientation of stereociliary bundles. We observed that genes associated with proliferation and differentiation (*cyclin d1*, *Jun*, *Lef1*, *Mapk8*) were down regulated two fold or more between these time points, while genes associated with antagonism of Wnt were up regulated by two fold or more (*Dkk3*, *Wif1*, *Wisp1*, *Sfrp3*) (Table 2). Between P0 and P6, the cochlea approaches maturity and the cells in the organ of Corti no longer respond to accumulation of β-catenin/canonical Wnt activation. Comparison of Ct values at these stages revealed that while *Wnt11* expression decreased 2.5 fold, both negative and positive regulators of Wnt are up regulated more than two fold (Table 2). This might reflect the maturation of the cochlear duct as it finishes its growth phase, and the nonsensory region undergoes cell rearrangements leading to thinning of the duct. Comparison of gene expression between P6 and P30 when the adult cochlea is fully formed showed up regulation of *Wnt2b*, *Dkk3*, *Wnt6* and *Dvl1*, these genes are likely necessary for homeostasis, while genes related to canonical Wnt signaling proliferation and planar cell polarity were down regulated (Table 2).

#### Expression of secreted Wnt signaling related molecules was dynamic

**Fig 2:**
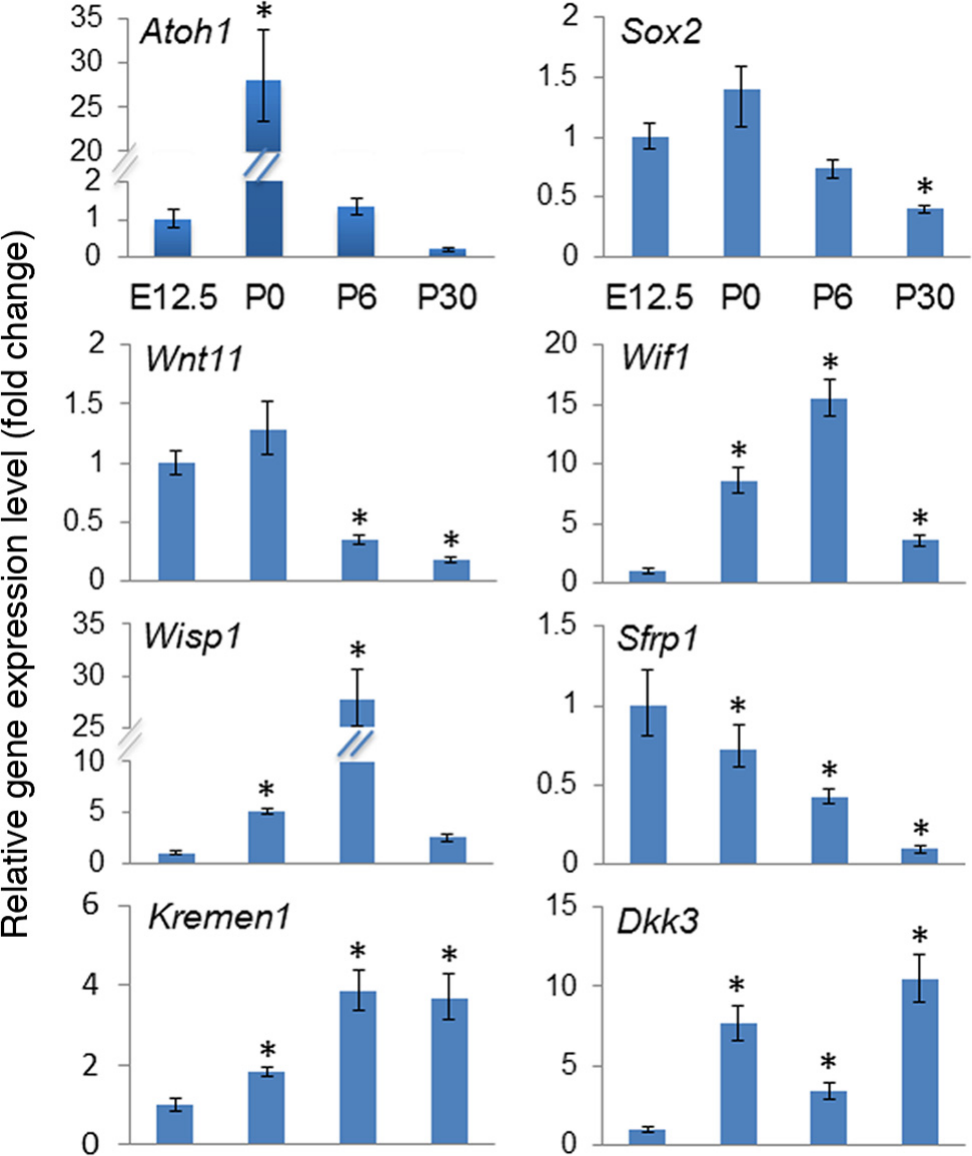
Expression of Wnt components is dynamic during postnatal maturation of the cochlea. Real-time quantitative PCR analysis of relative mRNA expression of selected Wnt signaling related genes, and cochlear markers Atoh1 and Sox2, on E12.5, P0, P6 and P30. Asterisks (*) mark a statistically significant fold change (student’s t-test, p<0.05) when compared to expression on E12.5. Each assay was performed in triplicate.

We identified five secreted molecules and one transmembrane receptor that showed a greater than two fold change in expression over time, and so were chosen for further investigation: *Wnt11*, *Wif1*, *Wisp1*, *Sfrp1*, *Dkk3* and *Kremen1*. These genes were of particular interest because, with the exception of Kremen1, they are secreted molecules that could be expressed at some distance from the sensory region or the vasculature of the cochlea. We used real time quantitative PCR (RT qPCR) to validate the fold change in the expression that we observed using the RT^2^ Profiler™ PCR Array assay (Fig 2). *Atoh1*, a gene required for hair cell differentiation during cochlear development, and *Sox2* a gene expressed in prosensory and supporting cells during cochlear development, were assayed as controls for cDNA quality. As expected, *Atoh1* expression increased dramatically from E12.5 to P0 and then was down regulated to a similar level as at E12.5 on P6, before being down regulated further in P30 cochleae (Fig 2). *Sox2* expression also behaved as expected, remaining at similar levels between E12.5 and P0 before declining by P6 and further by P30, as it is down regulated in hair cells and maintained only in supporting cells (Fig 2).

*Wnt11* demonstrated a similar expression profile to *Sox2*, expression was maintained from E12.5 to P0 and then declined through P6 and P30 (Fig 2). Wnt11 is often associated with PCP [23], but intriguingly has also been shown to activate canonical Wnt signaling in some contexts [23]. The presence of *Wnt11* as the sensory epithelium develops and then its decline as prosensory markers are down regulated is consistent with roles in either cell fate or PCP.

Next we compared expression levels of *Wif1*, a Wnt inhibitory factor that directly binds Wnts preventing them from interacting with the Wnt receptor complex [24] [1]. *Wif1* expression increased over 15 fold between E12.5 and P6, before being down regulated at P30 (Fig 2).

Wisp1 is an extracellular matrix bound molecule expressed in response to canonical Wnt signaling *in vitro*, and it is often observed in developing organs and tumors with high levels of Wnt signaling activity [25]. It functions in numerous pathways including acting as a mitogenic factor. *Wisp1* was expressed in a similar manner to Wif1, with a 25 fold increase in expression between E12.5 and P6 and a dramatic reduction by P30 (Fig 2). *Wisp1* expression in the cochlea peaked on P6, after Wnt signaling had ceased to be active in the sensory region, which could indicate a role for Wnt in expansion of the nonsensory epithelium or mesenchymal cells or Wnt signaling in the stria vascularis.

Sfrp1 is a secreted Wnt antagonist containing a Frizzled-like cysteine rich domain that can bind directly to Wnts, and likely the cysteine rich domain of Frizzleds [1]. In contrast to *Wnt11*, *Wif1* and *Wisp1*, *Sfrp1* was most highly expressed at E12.5 then declined ten fold between E12.5 and P30 (Fig 2). The decline in *Sfrp1* expression follows the trajectory of the decline of Wnt signaling in the sensory region. It is possible that Sfrp1 is involved in modulating canonical Wnt signaling during cell fate assignment of sensory and nonsensory cells in the sensory epithelium.

Lastly we examined expression levels of *Kremen1*, a transmembrane receptor, and *Dkk3*, a member of the Dkk family of secreted Wnt antagonists [1]. *Kremen1* expression increased four fold between E12.5 and P6 and was maintained at a high level up to P30 (Fig 2). This expression pattern is the inverse of Wnt reporter activity in the cochlea, suggesting a role for Kremen1 in modulating Wnt transduction as the cochlea matures. Finally, we assayed *Dkk3* expression (Fig 2). *Dkk3* expression increased more than five fold between E12.5 and P0; however, on P6, *Dkk3* expression was down regulated such that it was only around two fold higher than on E12.5. This reduction was consistent with a reduction in Wnt signaling activity on P6; however, by P30, Dkk3 expression had increased ten fold. This unusual pattern of expression was also reflected in the initial PCR assay, and warranted further investigation.

### Spatiotemporal expression analysis of secreted Wnt antagonists in the developing cochlea

Data generated by our PCR screens indicated that secreted antagonists of Wnt signaling *Sfrp1*, *Sfrp3* and *Dkk3*, were expressed concurrent with formation of the sensory epithelium. To determine whether these secreted signaling molecules were expressed in cells at the luminal surface of the cochlear duct, and thus free to diffuse to the sensory epithelium, we used *in situ hybridization* to define their exact spatial and temporal expression patterns.

#### Two members of the Sfrp family of Wnt antagonists were expressed in the developing cochlea

Based on data from the PCR screen we determined that members of the Sfrp family *Sfrp1*, *Sfrp2* and *Sfrp3* were expressed in the cochlea contemporaneously with formation of the sensory epithelium. *Sfrp1* and *Sfrp3* showed greater than two fold changes in expression between time points (Table 2) warranting investigation of their spatiotemporal expression.

**Fig 3:**
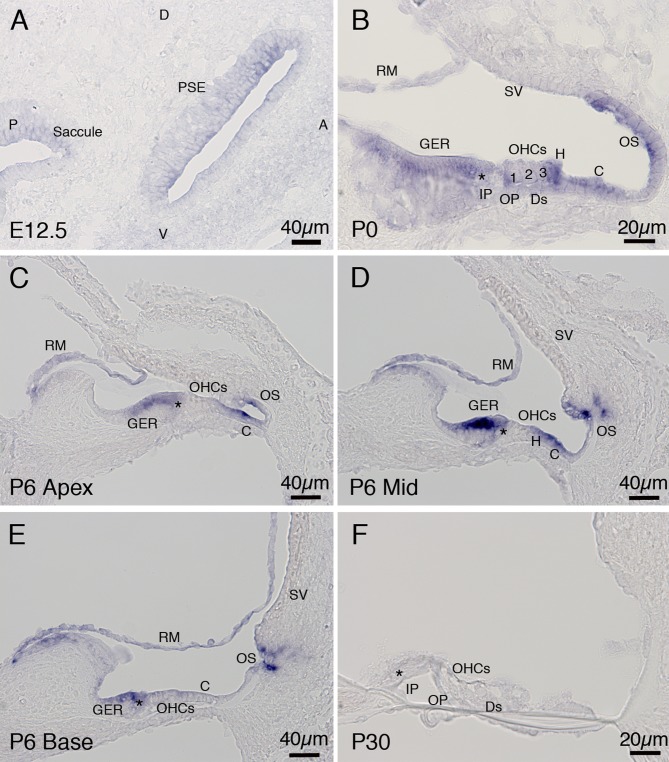
*In situ* hybridization analysis of *Sfrp1* mRNA expression in the developing cochlea. (A) A transverse section through the cochlea on E12.5. *Sfrp1* was broadly expressed in the prosensory epithelium. (B) A transverse section through the basal turn of the cochlea on P0. *Sfrp1* was expressed in the GER, LER, supporting cells and hair cells. (C, D, E) Transverse sections through the apical, mid and basal turn of a P6 cochlea. *Sfrp1* expression was detected in the GER and LER but was down regulated in the hair cells and supporting cells. (F) A transverse section through the cochlea on P30, revealed no detectable *Sfrp1*expression. Abbreviations: A: anterior; P: posterior; D: dorsal; V: ventral; PSE: prosensory epithelia; GER: greater epithelial ridge; LER: lesser epithelial ridge; inner hair cell (shown as asterisk *); IP: inner pillar cell; OP: outer pillar cell; OHC: outer hair cell; Ds: Deiters’ cells; H: Hensen’s cells; C: Claudius’ cells; SV: stria vascularis; OS: outer sulcus; and RM: Reissner’s membrane.

Consistent with our PCR data, *Sfrp1* was broadly expressed in the cochlea on E12.5 (Fig 3A). Later in development, on P0, *Sfrp1* expression was localized to the luminal surface of the floor of the cochlear duct, present in hair cells, supporting cells and the nonsensory cells of the greater epithelial ridge (GER) and lesser epithelial ridge (LER) (Fig 3B). The cochlea exhibits a developmental gradient, with the base being the most mature region and the apex the least mature. On P6 when, according to our RT qPCR data, *Sfrp1* begins to be down regulated, in the apical (Fig 3C) and mid basal turns (Fig 3D), *Sfrp1* expression remained throughout the floor of the duct, including the supporting cells, but not in the hair cells. In the most developmentally advanced region, the base, while expression of *Sfrp1* remained high in the GER, expression in the LER was down regulated and *Sfrp1* was not detected in hair cells and supporting cells (Fig 3E). As expected, on P30, when *Sfrp1* was down regulated ten fold, shown by RT qPCR, we observed no *Sfrp1* signal in the cochlear duct (Fig 3F).

**Fig 4:**
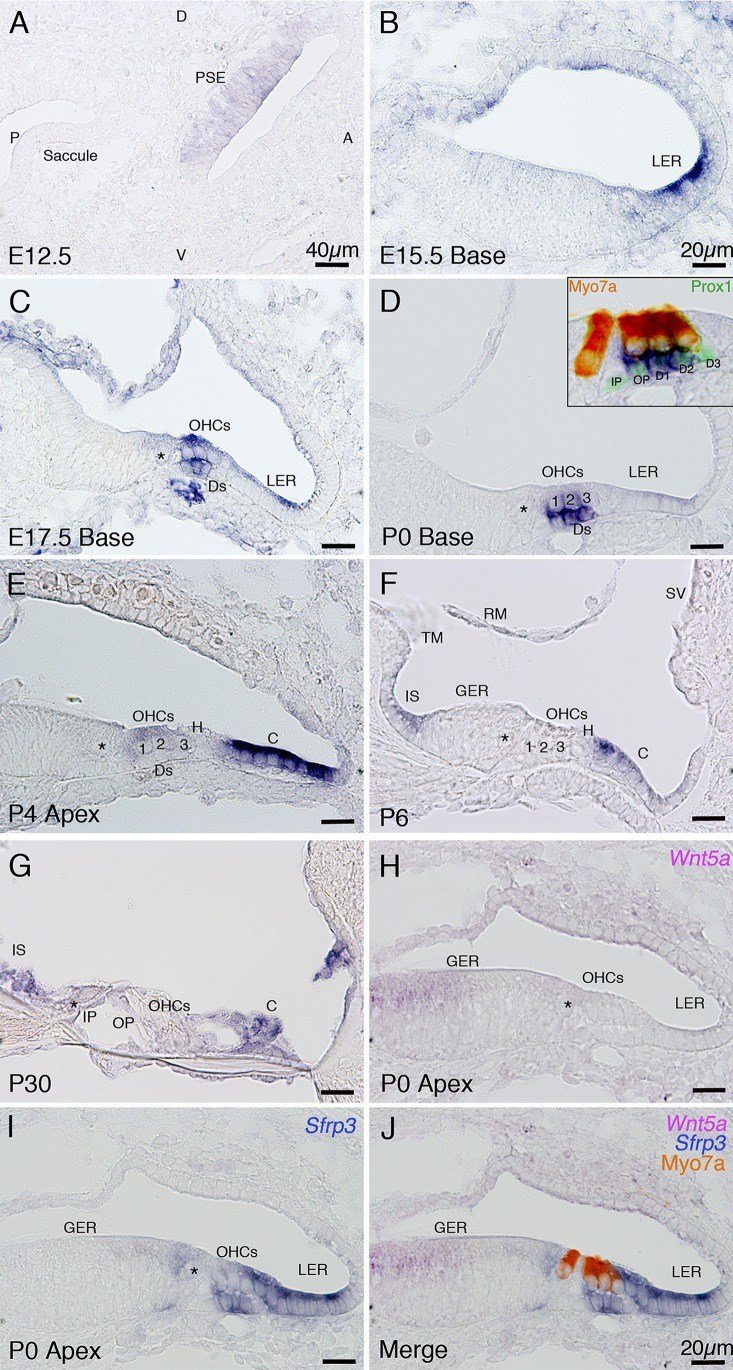
*In situ* hybridization analysis of *Sfrp3* mRNA expression in the developing cochlea. (A) Transverse section through the cochlea on E12.5. *Sfrp3* was expressed in the prosensory epithelia of the cochlea. (B) Transverse section through the basal turn on E15.5, *Sfrp3* expression was detected in the LER. (C) Transverse section through the basal turn on E17.5, *Sfrp3* expression was localized to the LER, outer hair cells and Deiters’ cells. (D) Transverse section through the basal turn on P0. *Sfrp3* expression was maintained in the outer hair cells and Deiters’ cells. Inset image shows a high magnification view the image in D, counterstained by immunofluorescence for hair cell marker Myo7a (red) and supporting cell marker, Prox1 (green), *Sfrp3* expression is detected in the outer pillar cell, the first and second Deiter’s cells but not in the inner pillar or third Deiters’ cell. (E) Transverse section through apical turn on P4. *Sfrp3* was expressed in the outer hair cells and Claudius’ cells. (F) Transverse section through the basal turn on P6. Sfrp3 expression was restricted to the inner sulcus and Claudius’ cells. (G) A transverse section through a P30 cochlea. *Sfrp3* expression was maintained in Claudius cells and the inner sulcus. (H) A transverse section through the apical turn of the cochlea on P0. *Wnt5a* mRNA expression localizes to the GER. (I) Transverse section adjacent to section used in (H). *Sfrp3* expression was detected at high levels in the sensory epithelium and the LER. (J) Image (H) overlaid with Image (I), demonstrating that *Sfrp3* and *Wnt5a* are reciprocally expressed. Hair cells were labeled by Myo7a (red). Abbreviations: A: anterior; P: posterior; D: dorsal; V: ventral; PSE: prosensory epithelium; GER: greater epithelial ridge; LER: lesser epithelial ridge; inner hair cell (shown as asterisk *); IP: inner pillar cell; OP: outer pillar cell; OHC: outer hair cell; Ds: Deiters’ cells; H: Hensen’s cells; C: Claudius’ cells; SV: stria vascularis; and RM: Reissner’s membrane.

*Sfrp3* was also expressed in the cochlear prosensory epithelium at E12.5 (Fig 4A). *Sfrp3* expression has previously been reported in the LER in the base at E14.5 [13]; in agreement with this result we observed *Sfrp3* expression in the LER of the basal turn on E15.5 (Fig 4B). Surprisingly, on E17.5 in the basal turn, in addition to the LER, *Sfrp3* expression was clearly detected in the OHCs and Deiters’ cells (Fig 4C). On P0, the expression in the LER persisted at relatively high levels in the least mature apical turn (Fig 4I), but was down regulated at the basal turn. This was in contrast to its expression in the hair cells and supporting cells where expression remained at high levels (Fig 4D). Expression of *Sfrp3* in the sensory epithelium persisted in the apical turn on P4 (Fig 4E); however, *Sfrp3* was expressed at far higher levels in the LER compared to the sensory epithelium (Fig 4E). This is a reversal of the expression pattern in the P0 base where expression of *Sfrp3* was higher in the sensory epithelium than the LER (Fig 4D). Notably *Sfrp3* was not found to be expressed in the inner pillar cells or third row of Deiters’ cells (Fig 4D, inset). On P6, *Sfrp3* was expressed strongly in cells of the LER and in cells of the inner sulcus, but was absent in the sensory epithelium (Fig 4F), this expression pattern persisted on P30 (Fig 4G). Development of the sensory epithelium progresses in a basal-apical gradient, this is reflected in the decreasing levels of expression of *Sfrp3* in the hair cells and supporting cells; however, expression of *Sfrp3* in the LER appears to follow an apical-basal gradient of expression being expressed in the apex and expanding to the base. Previous work has shown that application of recombinant Sfrp3 protein resulted in a similar phenotype to loss of *Wnt5a*, causing PCP defects in the stereociliary bundles, and failure of the sensory epithelium to undergo convergent extension mediated cell rearrangements [13]. Our *in situ* hybridization expression analysis of *Sfrp3* is consistent with a role regulating tissue polarity and in elongation of the cochlear duct based on expression in LER of the apex. Furthermore, we determined that on P0, *Wnt5a* is expressed in a complementary pattern to *Sfrp3* (Fig 4H, 4I and 4J).

The expression of secreted Sfrp family Wnt antagonists in the cochlea concurrent with formation of the sensory epithelium mirrors the activity of canonical and non-canonical PCP Wnt signaling. On E12.5 both *Sfrp1* and *Sfrp3* are expressed throughout the floor of the cochlear duct, and then later in the nonsensory regions flanking the sensory epithelium, and the sensory epithelium itself. This is suggestive of a role during Wnt mediated specification of presumptive hair cells, and as in the case of Sfrp3, a role in modulating the gradient of secreted Wnt5a during PCP mediated organization of the sensory epithelium. Recent reports have placed the two other members of the Sfrp (Sfrp4 and 5) family in the cochlea [26]. Similarly to Sfrp3, both of these secreted Wnt inhibitors disrupt orientation of stereociliary bundles, suggesting an inhibitory role in PCP signaling [26]. In regions where hair cell polarity was disturbed there also appeared to be additional hair cells. This could be attributed to failure of the cochlear duct to expand along the apico-basal axis leading to disorganization of the sensory region, or ‘bunching’ of the hair cells [26]. It is also possible that these Sfrp molecules activate the canonical Wnt signaling pathway [27]. Sfrp4 is a splice variant of *Frizzled 4* [27], which is expressed continuously in the sensory epithelium and would likely respond to stimulation with Sfrp4. We did not detect *Sfrp4* expression prior to P6. *Sfrp4* was expressed at relatively high levels on P6 and declined by P30. While it is clear that members of the Sfrp family are involved in modulation of Wnt mediated PCP, it remains to be determined whether they act as canonical Wnt antagonists, or canonical Wnt activators, in the inner ear. Additional study of individual Sfrp molecules and their effect on sensory epithelium development is required to unravel this question.

#### Localization of Dkk3 expression to distinct populations of cells dependent on developmental stage

**Fig 5:**
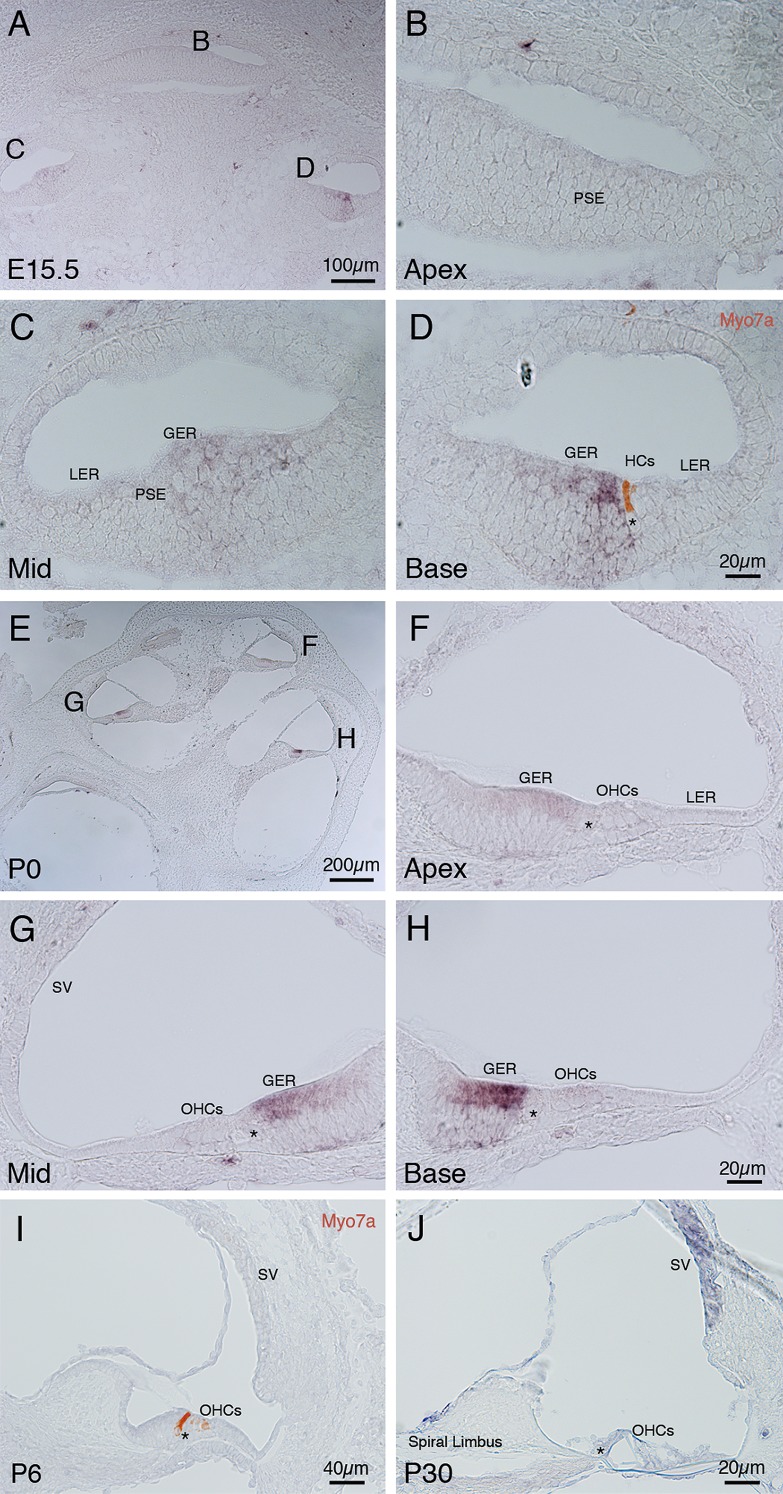
*In* situ hybridization analysis of *Dkk3* mRNA expression in the developing cochlea. (A) Transverse section through the cochlea on E15.5. (B-D) High magnification views of the apical, mid and basal turns of an E15.5 cochlea. (B) *Dkk3* expression was not observed. (C) *Dkk3* was detected in the GER. (D) *Dkk3* was detected in the GER adjacent to a single inner hair cell (labeled in red with hair cell marker Myo7a). (E) Transverse section through a cochlea on P0. *Dkk3* was detected in all three turns of the cochlea. (F-G) High magnification views of the apical, mid and basal turns. (F) *Dkk3* was detected at low levels in luminal cells of the GER. (G, H) *Dkk3* was detected at high levels in the GER. (I) Transverse section through a P6 cochlea. Dkk3 expression was down regulated. Hair cells were labeled by Myo7a (red). (J) Transverse section through a P30 cochlea showing *Dkk3* expression at the inner edge of spiral limbus and broadly in the stria vascularis. Abbreviations: PSE: prosensory epithelium; GER: greater epithelial ridge; LER: lesser epithelial ridge; HC: hair cell; Asterisk (*): inner hair cell; OHC: outer hair cell; SV: stria vascularis.

The dynamic expression pattern displayed by *Dkk3*, a secreted Wnt antagonist [1], suggested two discrete phases of *Dkk3* activity. In order to investigate this possibility we used *in situ* hybridization to precisely localize *Dkk3* expression. On E15.5 the three turns of the cochlea shown by a transverse section, represent the immature apex, and more advanced regions in the mid base and base (Fig 5A). We did not detect expression in the most immature apical turn (Fig 5A and 5B); however, *Dkk3* was detected by RT qPCR on E12.5 (Fig 2), it is possible that the limitations of DIG-labeled probes prevent visualization of such small transcript levels. As development of the sensory epithelium progressed, *Dkk3* expression initiated in the GER adjacent to the prosensory epithelium in the mid turn (Fig 5C), and was most strongly expressed in the GER adjacent to developing inner hair cells in the basal turn (Fig 5D).

We then localized *Dkk3* expression on P0, when mRNA transcripts were detected at their first peak (Fig 2). *Dkk3* expression was maintained in all three turns of the cochlea (Fig 5E, 5F, 5G and 5H). In the least mature, apical, turn, *Dkk3* expression was detected in the GER bordering the sensory epithelium (Fig 5F). In the more advanced mid turn, *Dkk3* expression remained in the GER, and was more intensely stained than in the apex (Fig 5G). In the most developmentally mature turn, the base, *Dkk3* expression was at its strongest (Fig 5H).

On P6, when according to our RT qPCR data *Dkk3* was down regulated, *Dkk3* was no longer strongly expressed in the GER, or any other cochlear structure (Fig 5I). Though *Dkk3* expression had declined in P6 tissue, expression was again detected in the stria vascularis and the inner edge of the spiral limbus at P30 (Fig 5J).

Previously, it has been reported that *Dkk3* expression is up regulated in the GER proximal to the sensory epithelium in response to deletion of the Notch component pRb [28]. In addition to *Dkk3*, several other Wnt related genes were up regulated (including *Tcf7l1*), leading the authors to suggest that *Dkk3* expression was elevated in order to prevent cells of the sensory epithelium from responding to Wnt signaling [28]. Furthermore, our previous work has shown that accumulation of β-catenin is necessary in order for hair cells to differentiate [10] [11], and that secreted Wnt signaling related molecules influence the expansion of the hair cell domain [11, 19]. Together this indicates that Dkk3 may have an inhibitory effect on inner hair cell differentiation co-incident with formation of outer hair cells.

Spatio-temporal localization of Wnt related gene expression has been performed in the developing chicken inner ear, revealing the presence of numerous secreted Wnts, Wnt receptors and Wnt antagonists [14]. Our screen indicates that there is some conservation of Wnt components across chicken and mouse during early stages of cochlear development: E12.5 in mouse and E4/5 in chicken. In common with the mouse during specification of the sensory region, *Wnt 4*, *5a*, *7a*, *9a* and *11* were expressed in the chicken cochlea—basilar papilla; however, Wnt 6 was expressed in chicken on E4/5 but not detected in the mouse cochlea until P0 [14]. The conservation between Wnt expression during the early phase of sensory specification is particularly noteworthy given that a Wnt responsible for activating canonical Wnt during hair cell differentiation has not yet been identified. *Frizzleds 1*, *2*, *4*, *7* and *8* were detected in both chicken and mouse but *Frizzled 10* was only found in chicken [14], and *Frizzleds 3*, *5* and *9* only in mouse. Of the Wnt antagonists, *Sfrp1*, *2* and *3* were expressed in the chicken and mouse inner ear during sensory specification; however, *Dkk3* expression was not reported in the chicken. In chicken, Sfrp1 was detected in the nonsensory region of the cochlear duct on E5-7 and E10-15 [14]; this was in contrast to *Sfrp1* expression in the mouse cochlea, where it initiated in the prosensory region of the cochlear epithelium, and was maintained in the hair cells, supporting cells, the GER and the LER throughout development. *Sfrp3* was expressed in the prosensory region of the developing basilar papilla and the nonsensory lateral wall on E4-5, by E5-7 *Sfrp3* was restricted to the extreme proximal and distal regions of the basilar papilla and the nonsensory region [14]. This was similar to the expression we observed in the mouse and suggestive of a common role in modulating organizational cues involved in extension of the cochlear duct and directionality of hair cells.

In conclusion, we have determined the expression of 72 Wnt signaling related genes, comprising intracellular and extracellular components of both canonical and non-canonical Wnt signaling. Furthermore, many of these genes are expressed dynamically as the cochlea matures. Our results are in agreement with previous reports of the presence of Frizzled receptor expression, and we identify multiple Wnt signaling related genes that have not been characterized in the mammalian ear. We show that secreted Wnt antagonists are expressed in the GER and LER, demonstrating the importance of screening the nonsensory tissue when investigating paracrine signaling in the cochlea. The potential of genes involved in the developmental phase of Wnt signaling to regenerate the sensory epithelium, represents a significant therapeutic avenue to restore hearing. Better understanding of the molecular context in which Wnt signaling takes place in the cochlea will lead to better approaches to harnessing Wnt mediated regeneration in the ear.
